# Biparametric MRI-based radiomics classifiers for the detection of prostate cancer in patients with PSA serum levels of 4∼10 ng/mL

**DOI:** 10.3389/fonc.2022.1020317

**Published:** 2022-12-05

**Authors:** Yangbai Lu, Binfei Li, Hongxing Huang, Qu Leng, Qiang Wang, Rui Zhong, Yaqiang Huang, Canyong Li, Runqiang Yuan, Yongxin Zhang

**Affiliations:** ^1^Department of Urology, Zhongshan City People’s Hospital, Zhongshan, Guangdong, China; ^2^Department of Anesthesiology, Zhongshan City People’s Hospital, Zhongshan, Guangdong, China; ^3^Department of Magnetic Resonance Imaging, Zhongshan City People’s Hospital, Zhongshan, Guangdong, China

**Keywords:** magnetic resonance imaging, prostate cancer, PI-RADS, radiomics, machine learning

## Abstract

**Purpose:**

To investigate the predictive performance of the combined model by integrating clinical variables and radiomic features for the accurate detection of prostate cancer (PCa) in patients with prostate-specific antigen (PSA) serum levels of 4-10 ng/mL.

**Methods:**

A retrospective study of 136 males (mean age, 67.3 ± 8.4 years) with Prostate Imaging-Reporting and Data System (PI-RADS) v2.1 category ≤3 lesions and PSA serum levels of 4-10 ng/mL were performed. All patients underwent multiparametric MRI at 3.0T and transrectal ultrasound-guided systematic prostate biopsy in their clinical workup. Radiomic features were extracted from axial T2-weighted images (T2WI) and apparent diffusion coefficient (ADC) maps of each patient using PyRadiomics. Pearson correlation coefficient (PCC) and recursive feature elimination (RFE) were implemented to identify the most significant radiomic features. Independent clinic-radiological factors were identified *via* univariate and multivariate regression analyses. Seven machine-learning algorithms were compared to construct a single-layered radiomic score (ie, radscore) and multivariate regression analysis was applied to construct the fusion radscore. Finally, the radiomic nomogram was further developed by integrating useful clinic-radiological factors and fusion radscore using multivariate regression analysis. The discriminative power of the nomogram was evaluated by area under the curve (AUC), DeLong test, calibration curve, decision curve analysis (DCA), and clinical impact curve (CIC).

**Results:**

The transitional zone-specific antigen density was identified as the only independent clinic-radiological factor, which yielded an AUC of 0.592 (95% confidence interval [CI]: 0.527-0.657). The ADC radscore based on six features and Naive Bayes achieved an AUC of 0.779 (95%CI: 0.730-0.828); the T2WI radscore based on 13 features and Support Vector Machine yielded an AUC of 0.808 (95%CI: 0.761-0.855). The fusion radscore obtained an improved AUC of 0.844 (95%CI: 0.801-0.887), which was higher than the single-layered radscores (both P<0.05). The radiomic nomogram achieved the highest value among all models (all P<0.05), with an AUC of 0.872 (95%CI: 0.835-0.909). Calibration curve showed good agreement and DCA together with CIC confirmed the clinical benefits of the radiomic nomogram.

**Conclusion:**

The radiomic nomogram holds the potential for accurate and noninvasive identification of PCa in patients with PI-RADS ≤3 lesions and PSA of 4-10 ng/mL, which could reduce unnecessary biopsy.

## Introduction

Prostate cancer (PCa) is among the most common malignancies in the male population and is a major global health problem (1). It is crucial for patients to be diagnosed with PCa as earlier as possible while reducing unnecessary biopsies. Currently, prostate-specific antigen (PSA) and multiparametric magnetic resonance imaging (mpMRI) both play essential roles in PCa screening and the selection of suitable candidates for biopsy (2, 3). However, PSA-based screening has several challenges including an increased false positive rate, the inability to detect PCa with random biopsy, multifocality in PCa, and the molecular heterogeneity of PCa (4). Around 70% of patients with PSA levels of 4-10 ng/mL, which is a diagnostic gray zone, may undergo unnecessary biopsy (5). mpMRI is a common non-invasive imaging technology applied in the detection and diagnosis of PCa, identification of aggressive disease, as a triage test before biopsy, targeting biopsy, and active surveillance of patients after a negative biopsy (6). However, mpMRI images may contain clinically valuable information related to tumor heterogeneity and biological characteristics, which may be difficult for radiologists to interpret in clinical practice (7), which calls for more advanced methods.

Radiomics is a novel tool that involves extracting quantitative features from medical images using computational algorithms such as machine learning, which can identify new biomarkers and assess the heterogeneity of the disease (8). Recently, radiomics and its combination with machine learning techniques have shown its promise in MRI-based PCa diagnosis, which was superior to Prostate Imaging-Reporting and Data System (PI-RADS) category (9–13). mpMRI protocol suggested by PI-RADS v2.0 includes T2-weighted imaging (T2WI), diffusion-weighted imaging (DWI) and the corresponding apparent-diffusion coefficient (ADC) maps, and dynamic contrast-enhanced (DCE) imaging (14). Despite several advancements in the diagnosis of PCa using mpMRI, it still suffers from relatively high cost, inconsistent image quality, moderate specificity, and inter-observer variability in result interpretation (15, 16). Some studies have shown that the incremental value of DCE imaging over the combination of T2WI and DWI in the diagnosis of PCa is modest (17–19). The PI-RADS v2.1 emphasizes that although DCE imaging is essential, its role in the determination of the PI-RADS category is secondary to that of T2-WI and DWI (20). Previous studies have shown that in those patients with PSA levels of 4-10 ng/mL, biparametric MRI (bpMRI) achieved better performance than conventional MRI and mpMRI with higher specificity in detecting PCa and reduced unnecessary biopsies (21–23). Accordingly, this study aimed to investigate the effectiveness of radiomic features extracted from bpMRI in the identification of PCa and to develop and validate a radiomic nomogram for PCa diagnosis in a subgroup of patients with PI-RADS ≤3 lesions and PSA serum levels of 4-10 ng/mL, providing a practical tool for accurate diagnosis and personalized treatment.

## Materials and methods

### Patients

**Figure 1:**
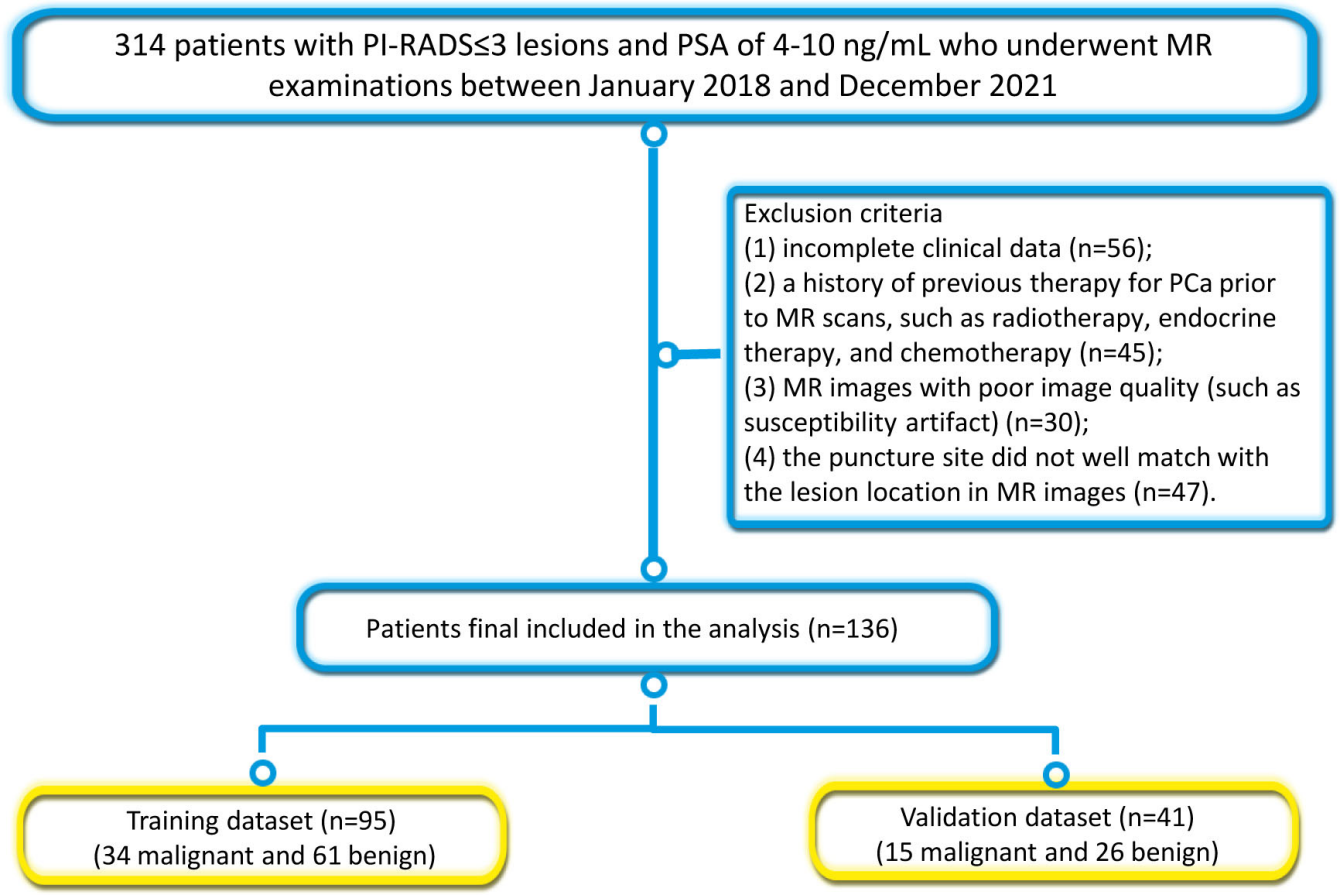
The patient recruitment pathway and the inclusion and exclusion criteria.

The Institutional Review Board approved this retrospective study and the requirement for written informed consent was waived. A total of 314 consecutive patients who underwent prostate MRI examination due to elevated PSA levels in our hospital between January 2018 and December 2021 were retrospectively reviewed. The inclusion criteria were as follows: (1) patients who received transrectal ultrasound-guided systematic prostate biopsy; (2) patients who underwent MRI scans within one week prior to biopsy; (3) patients with PI-RADS ≤3 lesions; and (4) patients with PSA serum levels of 4-10 ng/mL. The exclusion criteria were: 1) incomplete clinical data (n=56); 2) a history of previous therapy for PCa prior to MR scans, such as radiotherapy, endocrine therapy, and chemotherapy (n=45); 3) MR images with poor image quality (such as susceptibility artifact) (n=30); 4) the puncture site did not well match with the lesion location in MR images (n=47). Finally, 136 patients (mean age, 67.3 ± 8.4; range, 38-86 years) were included in this study. The patients were randomly divided into a training dataset (n=95; 34 PCa and 61 hyperplasia) and a validation dataset (n=41; 15 PCa and 26 hyperplasia). **Figure 1** illustrates the patient recruitment pathway and the inclusion and exclusion criteria.

### Clinical and radiological features

Baseline clinical variables were collected from the electronic medical record system, including age, total PSA (tPSA), free PSA (fPSA), and the ratio of fPSA to tPSA (fPSA/tPSA).

The length, width, and height of the entire prostate and transition zone (TZ) were measured on mpMRI by an experienced radiologist (with 11 years of experience in prostate MRI) and checked by a senior radiologist (with 20 years of experience in prostate MRI). The transverse diameter and anteroposterior diameter of the TZ, the transverse diameter and anteroposterior diameter of the entire prostate were measured on a horizontal section. The superoinferior diameters of the TZ and the entire prostate were measured on the sagittal plane. The prostate volume (PV) was measured at the boundary of the prostate capsule, and the transitional zone volume (TZV) was measured at the boundary of the fibrous layer of the TZ. The PV and TZV were calculated using the following ellipsoid volume formula: volume (ml) = (π/6) × anteroposterior diameter (cm) × transverse diameter (cm) × superoinferior diameter (cm). Peripheral zone volume (PZV) = PV - TZV. Prostate-specific antigen density (PSAD) = tPSA/PV, prostate transitional zone-specific antigen density (TZ-PSAD) = tPSA/TZV, and prostate peripheral specific antigen density (PZ-PSAD) = tPSA/PZV = tPSA/PV-TZV.

### MR imaging and image Interpretation

All patients were scanned on a 3.0T MR system (Achieva, Philips Medical Systems, Best, Netherlands) with a 16-channel Sense Torso XL coil. The MRI sequences included axial T1-weighted imaging, axial, coronal, and sagittal T2WI, axial DWI, pre-contrast axial fat- suppressed T1 high-resolution isotropic volume examination (THRIVE), and post-contrast axial breath-hold DCE that performed with fat-suppressed enhanced-THRIVE. The detailed acquisition parameters of routine MR sequences are shown in **Table 1**.

**Table 1:** The detailed acquisition parameters of MRI.

	T2WI	T1WI	DWI	eTHRIVE
TR/TE (ms)	3384/120	543/8	2787/61	3.1/1.8
Flip angle (°)	90	90	90	10
Slice thickness/gap (mm)	4	4	4	4/0
Acquisition time	02:55.9	01:52.4	01:54.3	01:50.6
FOV (mm)	230× 230	230× 230	250× 250	240× 240
Matrix	250× 250	250× 250	116×114	200× 200
Reconstruction matrix	0.57×0.57	0.57×0.57	1.12×1.12	0.58×0.58
Bandwidth (Hz/pixel)	1038.6	225.6	32.2	723.4
No. of excitations	1	1	4	1
B value (s/mm^2^)				0/1000

DWI, diffusion-weighted imaging; T2WI, T2-weighted imaging; T1WI, T1-weighted imaging; e-THRIVE, enhanced T1 high-resolution isotropic volume examination; TR/TE, repetition time/echo time; FOV, field of view.

A total of 20 periods of dynamic enhanced prostate scanning were performed, with a total scanning time of 2 min. The contrast agent was injected at the end of the first period. Gadodiamide (MEDRAD Healthcare, 0.2 mmol/kg body weight) was administrated *via* intravenously pumping (3.0 ml/s) followed by 20 ml of a saline flush at the same rate.

The PI-RADS v2.1 score for each case was evaluated by two radiologists (with 9 and 11 years of experience in PCa diagnosis, respectively), blinded to pathological data except for tumor location. Any discrepancy among the two observers was resolved by consulting with a third radiologist (with 20 years of experience in PCa diagnosis). The PI-RADS v2.1 scores were assessed on T2WI, DWI, and DCE-MRI images. If there were multiple lesions, the PI-RADS score was determined by the largest or most aggressive lesion.

### Tumor segmentation and volume of interest construction

**Figure 2:**
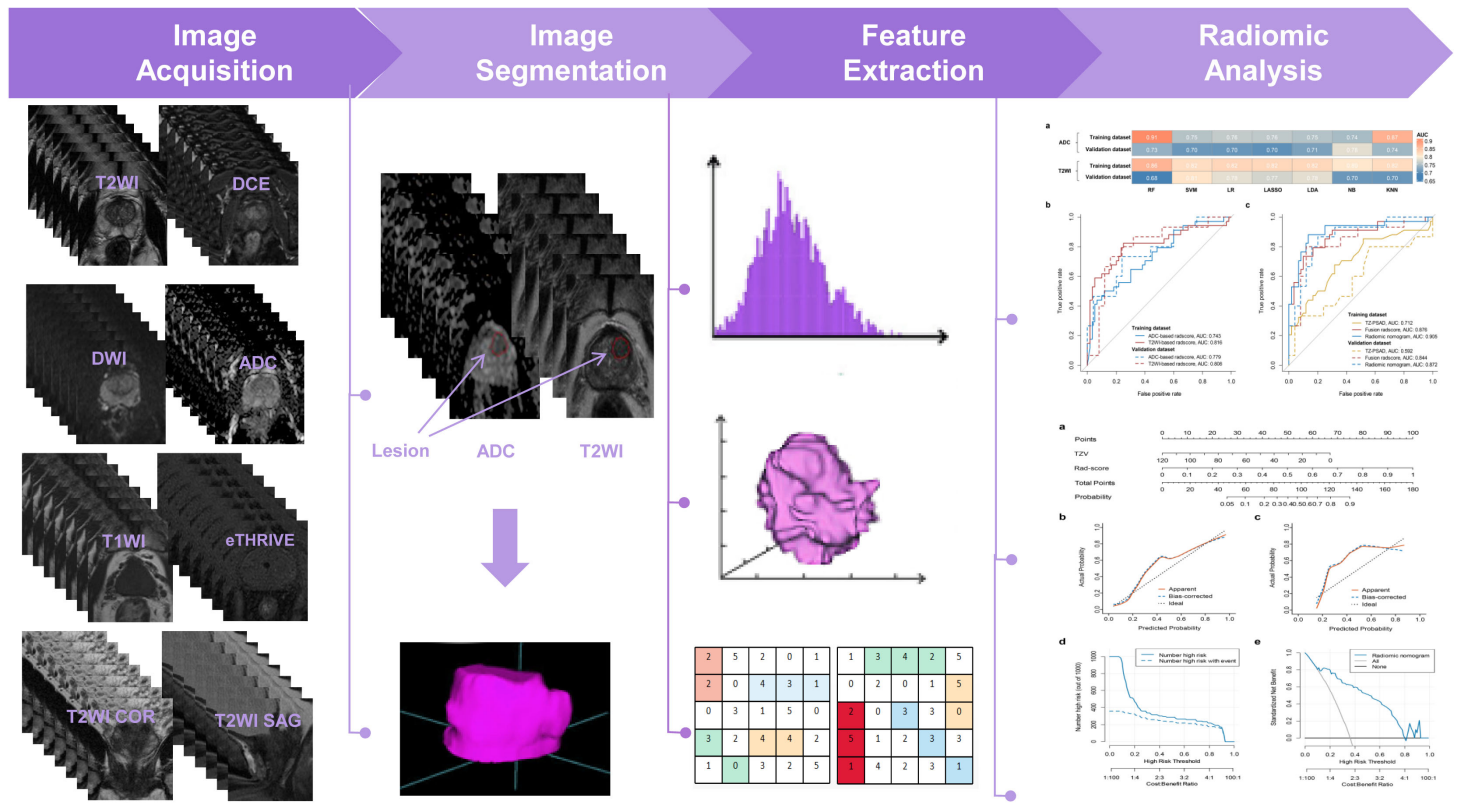
The workflow of this study.

**Figure 2** shows the workflow of this study. The manual segmentation of prostatic nodules was performed by a radiologist with 9 years of experience in PCa diagnosis using ITK-SNAP software (http://www.radiantviewer.com). The regions of interest (ROIs) were drawn by radiologists for radiomic analysis are confirmed by systematic biopsy cores. The lesions were manually outlined on each slice of the axial T2WI and ADC images separately, which covered the whole suspicious lesions and avoided the surrounding prostate capsule, peripheral blood vessels, seminal vesicle root, bleeding, calcification, and urethra. Then, ROIs were utilized to obtain volumes of interest (VOIs). The segmentation results were independently validated by a radiologist with 11 years of experience in PCa diagnosis to reduce potential bias.

### Image preprocessing

The N4 correction algorithm in the 3D Slicer (version 4.11.0; https://www.slicer.org) was used to remove the MRI offset field artifacts and reduce the inhomogeneity of the radio frequency field as well as the influence of the MR equipment itself. Then, the MRI gray value was normalized to reduce the gray difference between MRI sequences caused by different acquisition times and parameter settings, to ensure accurate and reliable radiomic analysis. Finally, the B-spline interpolation algorithm was used to resample the ROIs to a uniform size (1*1*1) for feature extraction.

### Feature extraction

The calculation and extraction of radiomic features were conducted consistently with the guideline of the Image Biomarker Standardisation Initiative (24). An open source radiomic package PyRadiomics v3.0 (25) (https://pypi.org/project/pyradiomics/) was applied to extract radiomic features from the original and filtered MR images (wavelet filter and Laplacian of Gaussian filter). Among them, the Log filter sigma parameter defines the texture roughness, and we set the sigma size to 1, 3, and 5 to obtain filtered images of different textures. For wavelet filtering, the bin width is set to 10. A total of 1130 radiomic features were extracted from each MR sequence, which could be divided into four groups: i) shape-based features (n=14), ii) first-order features (n=72), iii) texture features (n=300), and iv) wavelet features (n=744). Texture features included gray-level co-occurrence matrix (GLCM), gray-level size zone matrix (GLSZM), gray-level run length matrix (GLRLM), neighboring gray tone difference matrix (NGTDM), and gray-level dependence matrix (GLDM) features.

### Feature selection and radiomic score development

All radiomic features were standardized into a normal distribution with z-score normalization. A two-step feature selection process was employed by using several dimensionality reduction techniques. In the first step, we applied Pearson correlation coefficient (PCC) analysis to eliminate feature pairwise correlation and to ensure the features were independent of each other. One feature was selectively excluded from each pair with a correlation coefficient >0.99. In the second step, the remaining features were subsequently selected by the recursive feature elimination (RFE) algorithm. After that, seven machine learning classifiers were compared to build the best-performing T2WI- and ADC-based radiomic score (ie, radscore), including random forest (RF), support vector machine (SVM), logistic regression (LR), least absolute shrinkage and selection operator (LASSO), linear discriminant analysis (LDA), naive Bayes (NB), and K-nearest neighbor (KNN). Five-fold cross-validation was used for feature selection and classification algorithm optimization. The fusion radscore was obtained by linear fitting of T2WI- and ADC-based radscores using multivariate regression analysis in the training dataset.

### Radiomic nomogram establishment and assessment

Clinic-radiological variables with P<0.10 in univariate logistic regression were entered into multivariate logistic regression. The stepwise regression method was used to screen the variables in the multivariate regression model. Variables with P<0.05 were considered independent factors.

A radiomic nomogram was developed by integrating significant clinic-radiological variables in univariate regression analysis and radscore *via* multivariable logistic regression. Calibration curves of the nomogram and Hosmer–Lemeshow test were used to assess the agreement between prediction and actual observation. Moreover, we performed decision curve analysis (DCA) and clinical impact curve (CIC) to evaluate the net benefit of clinical decisions.

### Statistical analysis

All statistical analyses were done using R software (version 3.4.1; http://www.Rproject.org) and SPSS Statistics (version 26.0; IBM Corp., New York, USA). The distribution of continuous data was evaluated by the Kolmogorov-Smirnov test. Continuous and categorical variables were compared by t-test, Mann-Whitney U test, or Chi-square, if appropriate. The R package “glmnet” statistical software (R Foundation) was used to perform the modeling of classifiers. Other R packages were used as follows: “rms” package for multivariable logistic regression analysis and calibration curves, “pROC” package for receiver operating characteristic curve (ROC), “rmda” package for decision curve analysis (DCA), and clinical impact curve (CIC). ROC analysis was used to evaluate the classification performance of models, including area under the curve (AUC), sensitivity, specificity, and accuracy. The Delong test was used to compare the area under the curves (AUCs). P<0.05 was considered statistically significant.

## Results

### Patient characteristics

There were 57.4% of cases were in the TZ (benign 57.5% and malignant 57.1%). **Table 2** compares the clinic-radiological characteristics of benign and malignant prostatic nodules. The results showed that PV (P<0.001), TZV (P<0.001), PSAD (P=0.010), and TZ-PSAD (P=0.001) were statistically different between benign and malignant prostatic nodules. The clinic-radiological variables between the training and validation datasets were nonsignificant (all P>0.05).

**Table 2:** Comparison of clinic-radiological characteristics between benign and malignant prostatic lesions.

	All (n=136)	Benign (n=87)	Malignant (n=49)	P-value
Age, years	67 (62, 73)	67 (62, 71)	67 (61.5, 75)	0.678
TPSA	7.6 (6.08, 9.32)	7.4 (6.2, 9.2)	8 (5.81,10)	0.818
FPSA	1.1 (0.7, 1.7)	1.14 (0.8,1.72)	0.93 (0.54,1.86)	0.230
FPSA/TPSA	0.15 (0.1, 0.23)	0.16 (0.12, 0.22)	0.13 (0.09, 0.26)	0.355
PV	52.71 (35.39, 73.58)	63.01 (42.83, 77.69)	35.02 (26.95, 55.74)	<0.001
TZV	27.11 (11.77, 41.79)	33.69 (18.48, 52.19)	13.89(7.38,29.5)	<0.001
PZV	24.11(17.17,32.72)	27.04 (18.23, 34.39)	21.64 (14.43, 30.33)	0.097
PSAD	0.14 (0.1, 0.19)	0.13 (0.1, 0.16)	0.19 (0.1, 0.28)	0.010
TZ-PSAD	0.27 (0.16, 0.53)	0.22 (0.15, 0.42)	0.36 (0.22, 0.94)	0.001
PZ-PSAD	0.30 (0.21, 0.44)	0.28 (0.21, 0.42)	0.36 (0.19, 0.46)	0.607
PI-RADS				>0.999
1	2 (1.5)	1 (1.1)	1 (2.0)	
2	44 (32.4)	28 (32.2)	16 (32.7)	
3	90 (66.2)	58 (66.7)	32 (65.3)	

Unless otherwise indicated, continuous variables were presented as median (interquartile range, IQR), and categorical variables were presented as number (%).

TPSA, total prostate specific antigen; FPSA, free prostate specific antigen; PV, prostate volume; TZV, transitional zone volume; PZV, peripheral zone volume; PSAD, prostate specific antigen density; TZ-PSAD, prostate transitional zone-specific antigen density; PZ-PSAD, prostate peripheral specific antigen density; PI-RADS, Prostate Imaging Reporting and Data System.

### Clinical model

Univariate analysis of 11 clinic-radiological variables showed that PV (P=0.045), TZV (P=0.009), PSAD (P=0.008), and TZ-PSAD (P=0.001) were significantly associated with cancer (**Table 3**). The results of multivariate analysis showed that only TZ-PSAD (P=0.001) was an independent predictor of PCa (**Table 3**). The clinical model based on TZ-PSAD achieved an AUC of 0.712 (95%CI: 0.655-0.769) in the training dataset and 0.592 (95%CI: 0.527-0.657) in the validation dataset (**Table 4**).

**Table 3:** Univariate and multivariate logistic regression analyses of risk factors for cancer.

Variables	Univariate analysis		Multivariate analysis	
	OR (95%CI)	p	OR (95%CI)	p
Age, years	1.01 (0.96-1.06)	0.790		
PI-RADS				
2	Ref			
3	1.19 (0.50-2.84)	0.697		
TPSA	0.91 (0.76-1.10)	0.345		
FPSA	0.97 (0.68-1.38)	0.865		
FPSA/TPSA	0.57 (0.02-14.75)	0.735		
PV	0.98 (0.97-1.00)	0.045		
TZV	0.97 (0.95-0.99)	0.009		
PZV	1.01 (0.97-1.04)	0.749		
PSAD	1504.68 (6.53-346905.17)	0.008		
TZ-PSAD	17.91 (3.45-93.00)	0.001	17.91 (3.45-93.00)	0.001
PZ-PSAD	0.74 (0.12-4.72)	0.746		

OR, odds ratio; PI-RADS, Prostate Imaging Reporting and Data System; TPSA, total prostate specific antigen; FPSA, free prostate specific antigen; PV, prostate volume; TZV, transitional zone volume; PZV, peripheral zone volume; PSAD, prostate specific antigen density; TZ-PSAD, prostate transitional zone-specific antigen density; PZ-PSAD, prostate peripheral specific antigen density.

**Table 4:** The performance comparison of various models.

Dataset	Models	AUC (95%CI)	P	Sensitivity (%)	Specificity (%)	Accuracy
Training cohort	TZ-PSAD	0.712 (0.655-0.769)	<0.001	67.6	65.6	66.3
	ADC-based radscore	0.743 (0.690-0.796)	<0.001	64.7	68.9	67.4
	T2WI-based radscore	0.816 (0.765-0.867)	<0.001	82.4	73.8	76.8
	Fusion radscore	0.876 (0.837-0.915)	Ref.	85.3	73.8	77.9
	Radiomic nomogram	0.905 (0.870-0.940)	<0.001	88.2	85.2	86.3
Validation cohort	TZ-PSAD	0.592 (0.527-0.657)	<0.001	60.0	53.8	56.1
	ADC-based radscore	0.779 (0.730-0.828)	0.038	73.3	65.4	68.3
	T2WI-based radscore	0.808 (0.761-0.855)	<0.001	73.3	76.9	75.6
	Fusion radscore	0.844 (0.801-0.887)	Ref.	80.0	84.6	82.9
	Radiomic nomogram	0.872 (0.835-0.909)	0.002	73.3	84.6	80.5

AUC, area under the curve; TZ-PSAD, prostate transitional zone-specific antigen density; ADC, apparent-diffusion coefficient; T2WI, T2-weighted imaging.

### Comparison of single-layered radscores

**Figure 3:**
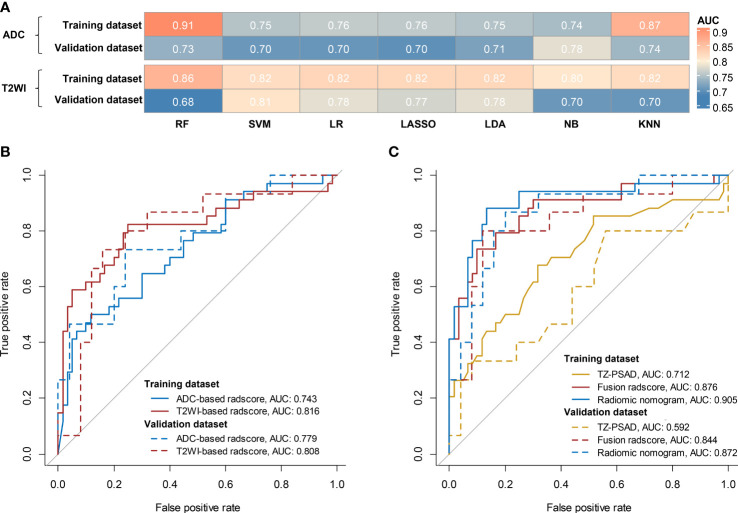
Diagnostic performance of models. **(A)** comparison of AUCs of different classifiers for ADC and T2WI, respectively; **(B)** ROC of the optimal single-layered radscore; **(C)** ROC of TZV, fusion radscore, and radiomic nomogram.

**Figure 4:**
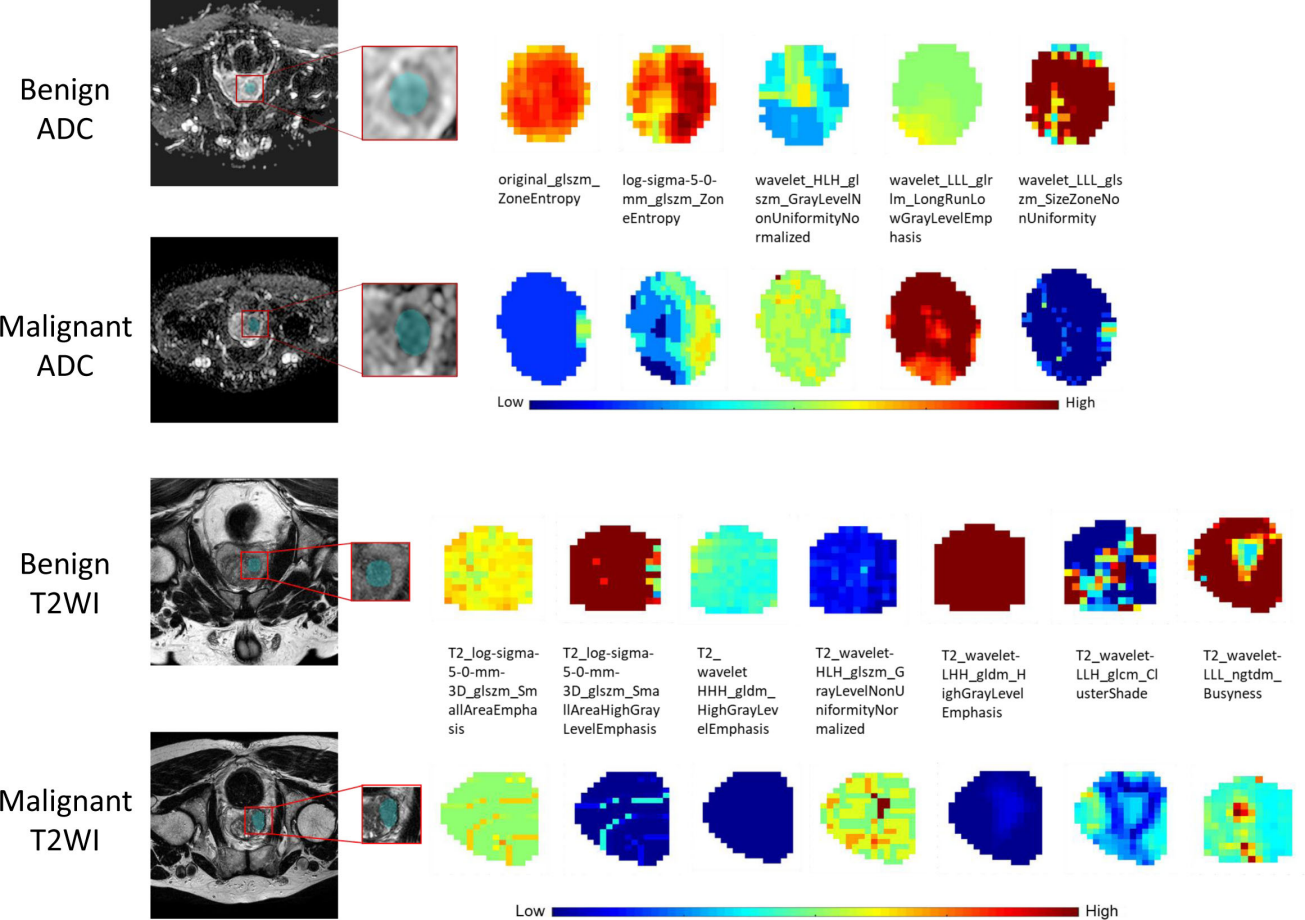
The visual feature maps of the selected texture features extracted from ADC- and T2WI images of benign and malignant cases.

For the ADC sequence, 679 radiomic features were retained after PCC analysis, and six features were finally selected (**Table 5**). The NB-based ADC radscore yielded the highest AUC of 0.743 (95%CI: 0.690-0.796) in the training dataset and 0.779 (95%CI: 0.730-0.828) in the validation dataset (**Table 4** and **Figure 3**). For the T2WI sequence, 681 features were retained after PCC analysis, and 13 features were finally identified (**Table 5**). The SVM-based T2WI radscore achieved the highest AUC of 0.816 (95%CI: 0.765-0.867) in the training dataset and 0.808 (95%CI: 0.761-0.855) in the validation dataset (**Table 4** and **Figure 3**). Compared with the single-layered radscores, the fusion radscore obtained higher performance (all P<0.05), with an AUC of 0.876 (95%CI: 0.837-0.915) in the training dataset and 0.844 (95%CI: 0.801-0.887) in the validation dataset (**Table 4** and **Figure 3**). **Figure 4** displays color-coded feature maps of the identified texture features derived from ADC- and T2WI images for qualitative visualization of the features. This figure demonstrates distinctive textures between benign and malignant cases.

**Table 5:** Radiomic features contained in the ADC-based and T2WI-based radiomic models.

**ADC**	
original_glszm_ZoneEntropy
log-sigma-5-0-mm-3D_firstorder_Range
log-sigma-5-0-mm-3D_glszm_ZoneEntropy
wavelet-HLH_glszm_GrayLevelNonUniformityNormalized
wavelet-LLL_glrlm_LongRunLowGrayLevelEmphasis
wavelet-LLL_glszm_SizeZoneNonUniformity
**T2WI**
log-sigma-1-0-mm-3D_firstorder_Kurtosis
log-sigma-5-0-mm-3D_glszm_SmallAreaEmphasis
log-sigma-5-0-mm-3D_glszm_SmallAreaHighGrayLevelEmphasis
wavelet-LLH_firstorder_Maximum
wavelet-LLH_glcm_ClusterShade
wavelet-LHH_gldm_HighGrayLevelEmphasis
wavelet-HLL_firstorder_Variance
wavelet-HLH_firstorder_Entropy
wavelet-HLH_glszm_GrayLevelNonUniformityNormalized
wavelet-HHL_firstorder_Skewness
wavelet-HHH_gldm_HighGrayLevelEmphasis
wavelet-LLL_firstorder_Minimum
wavelet-LLL_ngtdm_Busyness

ADC, apparent-diffusion coefficient; T2WI, T2-weighted imaging.

### Discriminative performance and clinical usefulness of radiomic nomogram

**Figure 5:**
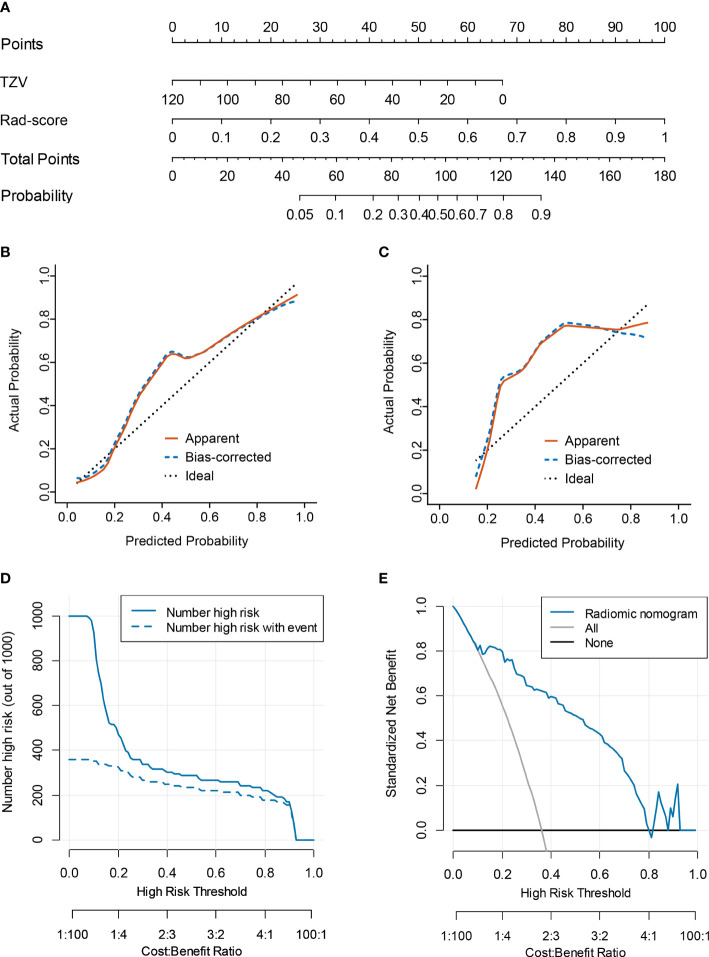
Nomogram performance for predicting probability of PCa. **(A)** nomogram; **(B)** calibration curve of training dataset; **(C)** calibration curve of validation dataset; **(D)** clinical decision curve; **(E)** clinical impact curve.

Multivariate logistic regression analysis of PV, TZV, PSAD, TZ-PSAD, and fusion radscore demonstrated that only TZV (P=0.011) and fusion radscore (P<0.001) were identified as two independent risk factors of cancer. The radiomic nomogram was developed based on TZV and radscore (**Figure 5**), the formula was as follows: nomogram score = -1.749 -0.033*TZV + 5.825*radscore. The radiomic nomogram achieved an AUC of 0.905 (95%CI: 0.870-0.940) in the training dataset and 0.872 (95%CI: 0.835-0.909) in the validation dataset, which was higher than TZ-PSAD and fusion radscore (all P values<0.05) (**Table 4** and **Figure 3**). The calibration curves of the radiomic nomogram demonstrated accepted agreement between prediction and actual observation (**Figure 5**), as verified by Hosmer-Lemeshow tests (training dataset: P=0.234; validation dataset: P=0.172). The DCA and CIC showed the clinical usefulness of the risk prediction nomogram.

## Discussion

This present study demonstrated that the radiomic model based on bpMRI achieved good performance in capturing the heterogeneity of prostatic diseases, which could be used to distinguish PCa from hyperplasia in a non-invasive way. The PSA-based screening strategy is not actable in patients with PI-RADS ≤3 lesions and PSA level of 4-10 ng/mL. However, the radiomic nomogram combining TZV and fusion radscore can individually predict the probability of PCa with high accuracy and good calibration and clinical utility.

In recent years, mpMRI has been increasingly applied for the qualitative evaluation of PCa (26, 27). The PI-RADS was proposed for better standardization of prostate MRI performance and image interpretation. PI-RADS v 2.1 in 2019 introduced the concept of bpMRI (T2WI and DWI) to simplify prostate MRI (20). Prostate MRI categorizes suspected PCa into low- and high-risk, with risk scores ranging from 1 to 5. PI-RADS hinges on the subjective judgment of radiologists, which is prone to inter-reader variability (28). A recent meta-analysis (29) showed varied inter-reader agreements of PI-RADS v2.1 and moderate inter-reader reliability (pooled k value of 0.65) among radiologists for whole gland and TZ lesions. Nonetheless, PI-RAS 2.1 has higher inter-reader reproducibility than version 2.0 (30). One of the most crucial challenges for clinicians is the occurrence of equivocal or PI-RADS 3 findings (31). While biopsy is recommended for PI-RADS 4 or 5 lesions, PI-RADS 3 lesions are considered borderline results with no definite recommendation or conclusion (20). Although PI-RADS 1 or 2 are not recommended for undergoing an imaging-guided biopsy, such an approach may miss a small portion of PCa due to the low cancer detection rate for PI-RADS 1 (6%; range, 0-20%) and PI-RADS 2 (9%; range, 5-13%) in the patient-level analysis (32). Additionally, whether patients with a serum PSA level of 4-10 ng/mL should be recommended for a biopsy is clinically challenging (33). In this current study, we focused on patients with both PI-RADS ≤3 lesions and PSA level of 4-10 ng/mL, which has not yet been reported in previous literature.

The clinical application of PSA has several limitations, leading to overdiagnosis and overtreatment (34, 35). Therefore, new methods for accurate risk stratification of prostatic nodules and reducing unnecessary biopsies are desirable to improve the management of PCa and patient prognosis. In recent years, non-invasive, cost-effective, high-accuracy liquid biopsy biomarkers such as DNA methylation have been developed for early diagnosis of PCa (36). However, liquid biopsy biomarkers still lack large-scale validation and have methodological and biological limitations, precluding implementation in clinical practice. Radiomics refers to the high-throughput mining of quantitative image features from standard-of-care medical images that enables to uncover of disease characteristics including biological property and tumor heterogeneity that fail to be appreciated by the naked eye (37). Radiomics provides a non-invasive way to evaluate PCa better than morphological visual interpretation. Li et al. (11) constructed a radiomic model based on mpMRI including T2WI, ADC, DWI, and DCE imaging and achieved an AUC of 0.86 when tested, improving diagnostic performance of PI-RADS v2.1 in PI-RADS categories 1-5 PCa. Qi et al. (33) found that mpMRI radiomics outperformed single sequence-based models; however, they did not explore the added value of DCE to T2WI and ADC. The results showed that the AUC of DCE model was lower than that of ADC and T2WI models (0.774 vs 0.853 vs 0.828). Thus, it seems that DCE may be difficult to bring significantly incremental value to bpMRI. Jing et al. (9) compared the combination of lesion segmentation and whole prostate segmentation on T2WI and DWI to establish the optimal radiomic methodology. The results showed that the radiomic model based on whole prostate T2WI and lesion DWI achieved the best performance in predicting clinically significant PCa, which was superior to PI-RADS scores (9). However, Montoya et al. (38) showed that bpMRI radiomics and kallikreins failed to outperform PI-RADS v2.1 scores and their combination did not achieve further performance improvement. Some studies compared different classifiers in the prospective diagnosis of prostate diseases based on mpMRI and found that the RF classifier performed better than other classifiers (39–42). Gui et al. (43) built a radiomic nomogram combing T2WI-based radiomic features and PSA yielded an AUC of 0.90 in the differential diagnosis of PCa and hyperplasia. Given that various deep-learning- and radiomics-based methods have been proposed for PCa classification, Castillo T et al. (44) compared the value of a deep-learning model with that of a radiomic model for the PCa diagnosis and concluded that the radiomic model was more accurate than a fully automated deep-learning model (AUC: 0.65-0.88 vs 0.44-0.73). Zhang et al. (45) showed that perilesional radiomic features could enhance the discrimination ability of the intralesional radiomic features. Lim et al. (46) indicated a moderate accuracy for T2WI (0.547) and ADC (0.684) for determining which PI-RADS category 3 lesions represent PCa. Considering the performance of radiomic model depends on feature selection and the employed machine learning algorithms, in this study, we carried out rigorous feature selection and compared seven machine learning classifiers to find the most suitable methods to build radiomic models, with an AUC of 0.84 in the validation dataset.

This study also has several limitations. First, this is a retrospective study performed in a single center with a relatively small sample size, which may indicate selection bias and low generalizability of the findings. Therefore, larger multicenter studies are warranted for reducing the effects of selection bias on model accuracy. Second, this study used manual segmentation rather than semi-automatic or automatic delineation, which was labored and time-consuming. Third, this study was unable to test a more advanced approach such as deep-learning for the prediction of PCa in such a population, which may show more merits and deserve further investigation. Finally, biological interpretability of texture features is lacking. Efforts to introduce biological meaning into radiomics are gaining traction in this field with distinct emerging approaches available, such as correlation with pathology features and biological function, radiology–pathology coregistration, and analysis of biological pathways or genomic correlations in humans or animals (47).

## Conclusion

In this study, we investigated the role of radiomic features derived from bpMRI (T2WI plus ADC) in the differentiation of PCa and hyperplasia. The combination of T2WI and ADC was superior to a single sequence in predicting PCa. The radiomic nomogram integrating TZV and radscore has the potential to accurate and noninvasive identification of PCa in patients with PI-RADS ≤3 lesions and PSA of 4-10 ng/mL, which could reduce unnecessary biopsy.

In the future, liquid biopsy diagnostic biomarkers can be added to improve the performance of MRI in early screening of PCa.

## Data availability statement

The raw data supporting the conclusions of this article will be made available by the authors, without undue reservation.

## Ethics statement

The studies involving human participants were reviewed and approved by Zhongshan City People’s Hospital. The ethics committee waived the requirement of written informed consent for participation.

## Author contributions

YBL and YXZ designed the study, collected and analyzed data, obtained the grant, wrote the manuscript; HXH, FBL, RQY assisted with data analysis and manuscript preparation. QL, QW, RZ, YQH, and CYL assisted with subject recruitment, data collection and analysis, manuscript review. All authors contributed to the article and approved the submitted version.

## Funding

This work was supported by grants from The Science and Technology Planning Project of Zhongshan City (No. 2020B1070, 2020B1073, 2019B1063), Major Project of Scientific Research Foundation, Zhongshan city people’s hospital (No.BG20228249), Leading Specialist Construction Project Department of Urology, Zhongshan City People’s Hospital (No.G330102097008), Leading Specialist Construction Project Center of Radiology, Zhongshan City People’s Hospital (No.T2020016).

## Conflict of interest

The authors declare that the research was conducted in the absence of any commercial or financial relationships that could be construed as a potential conflict of interest.

## Publisher’s note

All claims expressed in this article are solely those of the authors and do not necessarily represent those of their affiliated organizations, or those of the publisher, the editors and the reviewers. Any product that may be evaluated in this article, or claim that may be made by its manufacturer, is not guaranteed or endorsed by the publisher.
